# 112 Promoting Physical Activity in Workplaces: The Integral Role of Well-being Management

**DOI:** 10.1093/eurpub/ckae114.182

**Published:** 2024-09-26

**Authors:** Miia Malvela, Reetta Laakkonen, Ossi Aura, Tomi Hussi

**Affiliations:** Jyväskylä University Of Applied Sciences, Jyväskylä, Finland; Jyväskylä University Of Applied Sciences, Jyväskylä, Finland; KuntoBoss Oy, Helsinki, Finland; ThinkFellows Oy, Espoo, Finland

## Abstract

**Purpose:**

The primary purpose of the research was to find out how employers in Finland support physical activity at workplaces and how physical activity is related to the management of well-being at work. In the new government program in Finland, physical activity is integrated as a part of developing work ability and well-being at work.

**Methods:**

The Adults on the Move program conducted the Finnish barometer on physical activity at workplaces. The research was carried out by interviewing 300 employers representing five industries and 1100 employees. The role of the management practices on activity of worksite physical activity was analyzed using regression analysis. Indicators were formed from the data.

**Results:**

In the barometer, questions were related to physical activity as well as to the management of well-being at work. The basic decisions of well-being management are setting the goal, making the plan, and deciding the responsibility of the supervisors.

The results showed that the implementation of physical activity at workplaces is strongly linked to the quality of well-being management. When all three basic decisions of well-being management are implemented, the action to increase physical activity is carried out most effectively:

• Most diverse task-specific physical activity solutions are in use,

• Physical activity is taken into account in remote work,

• Physical activity suppors recovery,

• Physical activity is part of the cooperation with occupational health care,

• Physical activity is included in supervisor coaching and training and further in personnel development discussions.

**Conclusions:**

The level of well-being management is thus related to the implementation and diversity of physical activity at workplaces. Basic management decisions were more commonly made in large organizations. The management of small and medium-sized companies needs training on physical activity and well-being at work, so that the means of physical activity for the personnel would be optimal and high-quality. In this way, work ability and well-being at work are increased through physical activity.

**Funding:**

Adults on the Move Program, Ministry of Education and Culture.

